# Outcome of polycystic kidney disease patients on peritoneal dialysis: Systematic review of literature and meta-analysis

**DOI:** 10.1371/journal.pone.0196769

**Published:** 2018-05-22

**Authors:** Vincent Dupont, Lukshe Kanagaratnam, Mickaël Sigogne, Clémence Bechade, Thierry Lobbedez, Jose Portoles, Philippe Rieu, Moustapha Drame, Fatouma Touré

**Affiliations:** 1 Division of Nephrology, University Hospital of Reims, Reims, France; 2 Department of Research and Innovation, University Hospital of Reims, Reims, France; 3 Division of Nephrology, University hospital of Caen, Caen, France; 4 Division of Nephrology, University Hospital of Puerta de Hierro, Madrid, Spain; 5 Laboratory of Nephrology, UMR CNRS URCA 7369 (Matrice Extracellulaire et Dynamique Cellulaire, MEDyC), Reims, France; Kaohsiung Medical University Hospital, TAIWAN

## Abstract

**Background:**

Polycystic kidney disease (PKD) is the most frequent hereditary cause of chronic kidney disease. Peritoneal dialysis (PD) is often avoided for patients with PKD because of the suspected risk of mechanical and infectious complications. Only a few studies have analyzed the outcome of PKD patients on PD with sometimes conflicting results. The purpose of this meta-analysis was to investigate outcomes of patients with PKD treated by PD.

**Methods:**

A systematic review and meta-analysis were performed examining all studies which included “Polycystic kidney disease” and “Peritoneal dialysis” in their titles, excluding commentaries, letters to the authors and abstracts. PubMed, Embase, Google scholar and Scopus were searched to December 31^st^ 2017. The primary outcome was overall patient survival. Additional outcomes were PD technique survival, incidence of peritonitis and incidence of abdominal wall hernia.

**Results:**

9 studies published between 1998 and 2016 were included for analysis with a total of 7,197 patients including 882 PKD patients. Overall survival of PKD patients was found to be better compared to non-PKD patients (HR = 0.70 [95% CI, 0.54–0.92]). There were no statistical differences between PKD and non-PKD patients in terms of peritonitis (OR = 0.86 [95% CI, 0.66–1.12]) and technical survival (HR = 0.98 [95% CI, 0.83–1.16]). There was an increased risk of hernia in PKD patients (OR = 2.28 [95% CI, 1.26–4.12]).

**Conclusions:**

PKD is associated with a better global survival, an increased risk of abdominal hernia, but no differences in peritonitis rate or technical survival were found. PD is a safe dialysis modality for PKD patients. Properly designed controlled studies are needed to determine whether all PKD patients are eligible for PD or whether some specific criteria should be determined.

## Introduction

Polycystic kidney disease (PKD) is the most frequent hereditary cause of chronic kidney disease. [1,2]. Peritoneal dialysis (PD) is often avoided for PKD patients when it comes to the choice of renal replacement therapy. Indeed, enlarged kidneys and liver may reduce the peritoneal extensibility, leading to increased intraperitoneal pressure [3,4]. Higher prevalence of abdominal wall hernia, leaks, and diverticulitis-related peritonitis have been reported with PD administration in PKD patients [5]. These complications may directly impact on PD technique survival and on patients’ outcomes. However, only a small number of studies have been designed to analyze the incidence of these events and their relation with PD technical survival or global patient survival. The purpose of this review and meta-analysis was to investigate the outcome of patients with PKD treated by PD.

## Methods

### Study design

This systematic review with meta-analysis was conducted according to a prespecified protocol and was reported using Preferred Reporting Items for Systematic Reviews and Meta-Analyses (PRISMA) guidelines [6].

### Search strategy and selection criteria

A bibliographic search was performed from the inception to December 31^st^ 2017 in the following databases: Pubmed, Embase, Google scholar and Scopus. We also screened references of included articles to identify other potential studies. The search strategy, on the article title, was as follows: "polycystic kidney"[title] OR "polycystic kidney disease"[title] OR ADPKD[title] or "autosomic dominant polycystic kidney disease"[title]) AND "peritoneal dialysis"[title]. One author (VD) performed the full search strategy and removed duplicates.

### Study selection and data extraction

After eliminating duplicates, two authors (VD, MS) independently reviewed the titles and abstracts of all articles. Disagreements were resolved by consensus. Agreement between the two authors was assessed using the Kappa coefficient. After agreement, the full text of all articles designated for inclusion was obtained. Two authors (VD, MS) checked to ensure that all articles met the criteria for inclusion in this analysis, and then independently extracted the data into a standardized form. Extracted data were: study design, country, number of subject included, percentage of male, age, comorbidity (Charlson index, diabetes mellitus and hypertension), percentage of patients treated by automated peritoneal dialysis, transfer to hemodialysis, access to kidney transplantation, dialysis adequacy, hemoglobinemia, albuminemia, overall survival, PD technique survival defined as permanent cessation of PD therapy due to PD related complications, and occurrence of peritonitis or abdominal hernia. Study authors were contacted to obtain missing data. Studies were included if they presented at least two of the following parameters: overall survival, PD technique survival, incidence of peritonitis, and incidence of abdominal hernia. Studies were excluded if they presented any one or more of the following criteria: case report, case series, abstracts, commenters or letter to the editor, language other than French or English.

### Risk-of-Bias assessment

The quality of included studies was assessed independently by two researchers (VD, MS) using the Newcastle-Ottawa scale (NOS) for cohort studies [7]. The NOS consists of three quality parameters, namely selection, comparability, and outcome assessment. The NOS assigns a maximum of four points for selection, two points for comparability and three points for outcome. NOS scores of ≥7 were considered as high quality studies and 5–6 as moderate quality [8]. Disagreement was resolved by joint review of the manuscript to reach consensus. Publication bias was assessed using funnel plots and the Egger’s regression test if there were up to 10 eligible studies included in the meta-analysis [9,10].

### Statistical analysis

The primary outcome was overall survival. Secondary outcomes were: 1/ PD technique survival defined as permanent cessation of PD therapy due to PD related complications, (considering any other outcome as censored data), 2/ percentage of peritonitis and 3/ frequency of abdominal hernias. Extracted data were presented as number and percentage for qualitative variables, and as mean and standard deviation (or median and range) for quantitative variables. Heterogeneity between studies was assessed using the Cochran Q statistic and I^2^ test. A random effects model was used independently of the existence or absence of heterogeneity between the results of the studies because results of studies with different design and patients’ characteristics were pooled. For time to event outcomes, when hazard ratios (HR) were not specified, they were estimated according to the information presented in the paper [11]. PKD and non PKD patients were compared through random effects models weighted by the inverse-variance method to estimate pooled HR and odds ratios (OR) with 95% confidence intervals (CIs). Sensitivity analyses were performed. All analyses were performed using R version 3.1.2 (R Foundation for Statistical Computing, Vienna, Austria).

## Results

### Study characteristics

**Fig 1:**
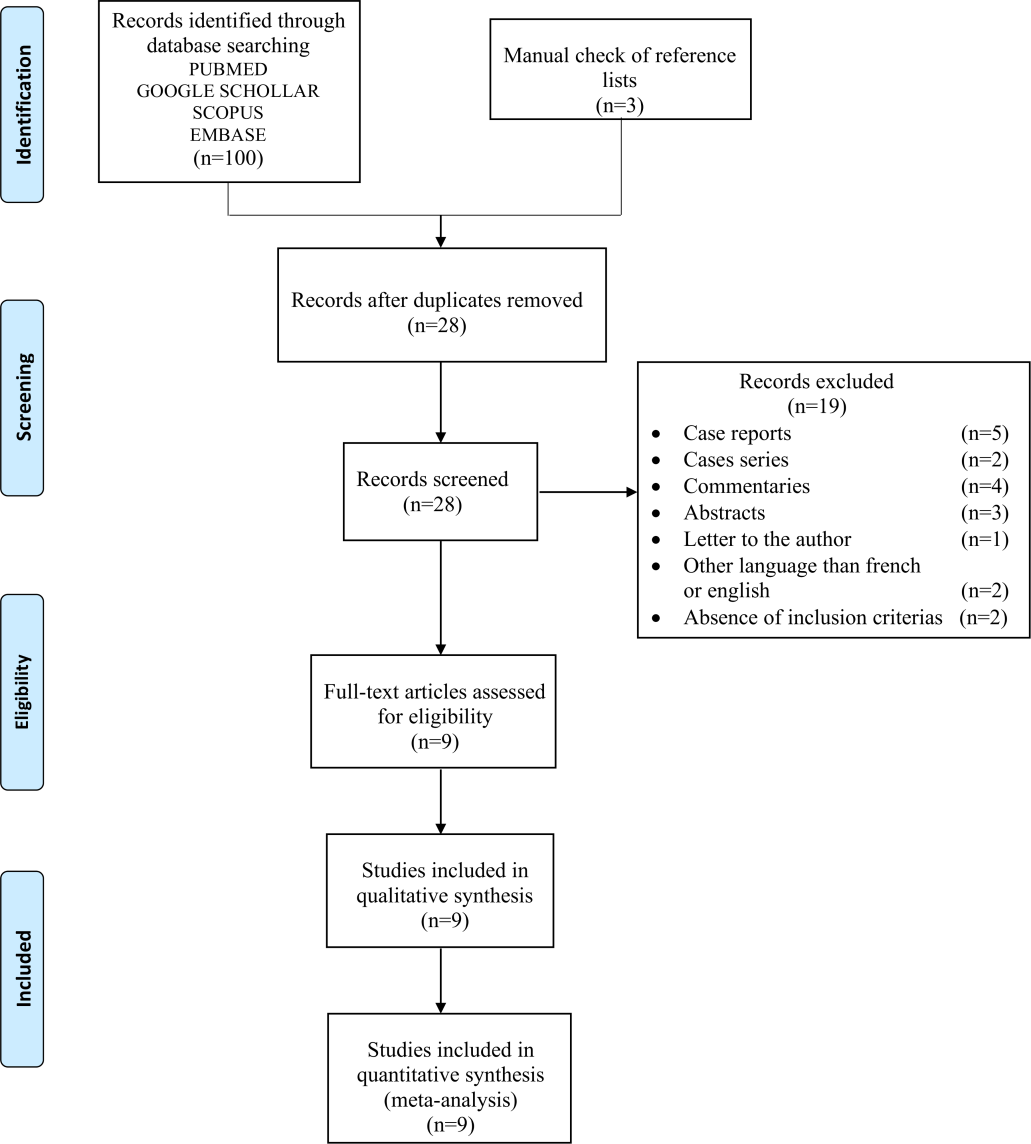
Literature search strategy and results.

Using the search strategy (Fig 1), we identified 9 eligible studies [12–20] presenting at least two outcome of interest. The agreement in selection of studies between the reviewers was excellent (κ = 1). All the studies included in the meta-analysis were considered as high quality studies (details showed in Table 1). Publication bias, using funnel plot and the Egger’s regression test, was not assessed as the number of studies included in this meta-analysis was less than 10 studies [9,10].

**Table 1:** Quality of the studies included in the meta-analysis assessed with Newcastle-Ottawa scale (n = 9).

Study, Year	Selection	Comparability	Outcome	NOS
**Jankowska et al, 2015**	****	**	***	9
**Janeiro et al, 2015**	****	**	***	9
**Yang et al, 2015**	****		***	7
**Lobbedez et al, 2010**	****	**	***	9
**Li et al, 2011**	****		***	7
**Kumar et al, 2008**	****		***	7
**Xie et al, 2016**	****		***	7
**Hadimeri et al, 1998**	****		***	7
**Koc et al, 2016**	****		***	7

NOS, *Newcastle-Ottawa Scale score*; the number of stars (*) corresponds to the number of complete items (maximum 4 items of Selection, *2 items for Comparability and 3 items for Outcome)*

General characteristics of the studies included in the meta-analysis are presented in Table 2. A total of 7,197 patients were included for analysis, including 882 PKD patients. Seven of the 9 studies were retrospective. In two of the 9 studies [14,20], PKD patients were significantly younger than non PKD.

**Table 2:** General characteristics of the studies included in the meta-analysis (n = 9).

Study, Year	Country	Study Design	n	n		Mean Age		*p*	Male (%)		Follow up (M)	Adjusted variables
				PKD+	PKD-	PKD+	PKD-		PKD+	PKD-		
**Jankowska et al, 2015**	Poland	Prospective cohort	1712	106	1606	62 (55–72)	60 (47–71)	0.04*	42.5	53.3	32	Age, sex, diabetes, hypertension
**Janeiro et al, 2015**	Spain	Prospective matched cohort	318	106	212	54.28 (±11.92)	53.43 (±16.43)	0.6	60	61.4	21	Age, CIS
**Yang et al, 2015**	Taiwan	Retrospective propensity-score matched cohort	556	139	417	53.4 (±14.7)	53.8 (±14.7)	0.78	51.8	54.9	32	-
**Lobbedez et al, 2010**	France	Retrospective cohort	4162	344	3818	56.2 (47.1–67.9)	71.1 (52.4–80.7)	-	48	59	17	Age, sex, CIS, center, type of assistance
**Li et al, 2011**	China	Retrospective matched cohort	126	42	84	57.3 (±12.0)	56.0 (±11.9)	0.6	64.2	46.4	42	
**Kumar et al, 2008**	England	Retrospective matched cohort	112	56	56	50.8 (±11.6)	50.3 (±11.5)	NS	45	45	37	
**Xie et al, 2016**	China	Retrospective matched cohort	60	30	30	52.5 (±11.0)	52.6 (±11.1)	0.962	60	60	27	
**Hadimeri et al, 1998**	Sweden	Retrospective cohort	52	26	26	57 (±11)	53 (±14)	0.002*	65	73	18	
**Koc et al, 2016**	Turkey	Retrospective cohort	99	33	66	35.4 (±13.1)	46 (±16.8)	-	39.4	53	150	

n, *number of subjects*; PKD+, *Patients with polycystic kidney disease*; PKD-, *Patients without polycystic kidney disease*; M, *Months (median)*; CIS, *Charlson Index Score*

*, p<0.05

Clinical characteristics of patients included are summarized in Table 3. Patients did not differ between the PKD and non PKD groups in terms of hypertension, access to kidney transplantation and transfer to hemodialysis. In one of the 9 studies [14], Charlson index was found to be higher in non PKD patients. Most of the patients were treated by continuous ambulatory peritoneal dialysis while only Yang et al. reported a higher prevalence of automated peritoneal dialysis in the PKD group [12].

**Table 3:** Clinical characteristics of the studies included in the meta-analysis (n = 9).

										Outcome					
Study, Year	Hypertension (%)		*p*	CIS		*p*	APD (%)		*p*	Kidney transplantation (%)		*p*	Transfer to hemodialysis (%)		*p*
	PKD+	PKD-		PKD+	PKD-		PKD+	PKD-		PKD+	PKD-		PKD+	PKD-	
**Jankowska et al, 2015**	83	83.9	0.9	-	-	-	45.3	44.4	0.9	26.4	18.6	0.04*	18.9	21.4	0.5
**Janeiro et al, 2015**	14.7	23	0.21	4.27 (±1.58)	5.27 (±2.5)	<0.001*	43.4	33.7	0.1	47.2	30.7	0.004*	17	20.3	NS
**Yang et al, 2015**	71.2	73.1	0.66	2.9 (±1.4)	3.0 (±1.4)	0.5	46.8	37.6	0.03*	9.4	9.8	0.87	24.5	22.5	0.64
**Lobbedez et al, 2010**	-	-	-	3 (3–5)	6 (3–7)	-	54.9	34.5	-	52	30	-	32	30	-
**Li et al, 2011**	95.2	91.7	0.5	4.6 (±1.6)	4.1 (±1.8)	0.1	-	-	-	9.5	11.9	-	9.5	11.9	-
**Kumar et al, 2008**	-	-	-	-	-	-	18.5	17.8	-	39	37	NS	30	25	NS
**Xie et al, 2016**	100	93.3	0.152	3.3 (±1.1)	3.1 (±1.0)	0.351	-	-	-	16.7	16.7	1	16.7	20	0.506
**Hadimeri et al, 1998**	-	-		-	-	-	0	0	-	60	41.2	-	25	29.4	-
**Koc et al, 2016**	-	-	-	-	-	-	42.4	47	0.36	30.3	16.6	-	30.3	28.8	-

PKD+, *Patients with Polycystic Kidney* Disease; PKD-, *Patients without Polycystic Kidney* Disease; CIS, *Charlson Index* Score; APD, *Automated Peritoneal* Dialysis

*, p<0.05

Biological characteristics of patients included are shown in Table 4. There was no difference in serum albumin level and dialysis adequacy in term of total weekly Kt/V urea between PKD and non PKD patients. In two studies [14,17], hemoglobin level was higher in PKD patients.

**Table 4:** Biological characteristics of the studies included in the meta-analysis (n = 9).

Study, Year	Hemoglobinemia [g/dL]		*p*	Albuminemia [g/L]		*p*	Kt/V		*p*
	PKD+	PKD-		PKD+	PKD-		PKD+	PKD-	
**Jankowska et al, 2015**	11.4 (±1.5)	11.2 (±1.7)	0.2	37.3 (±6.0)	36.0 (±7.3)	0.1	2.22 (±0.59)	2.33 (±0.71)	0.2
**Janeiro et al, 2015**	12.63 (±1.44)	11.96 (±1.49)	0.001*	-	-	-	2.68 (±0.65)	2.52 (±0.80)	0.1
**Yang et al, 2015**	-	-	-	-	-	-	-	-	-
**Lobbedez et al, 2010**	-	-	-	-	-	-	-	-	-
**Li et al, 2011**	10.4 (±1.7)	9.0 (±1.3)	<0.001*	32.0 (±5.9)	33.0 (±5.0)	0.4	1.93 (±0.39)	1.99 (±0.37)	0.4
**Kumar et al, 2008**	11.0 (±1.5)	10.6 (±1.9)	NS	39.8 (±3.9)	38.1 (±4.9)	NS	2.3 (±0.50)	2.1 (±0.4)	NS
**Xie et al, 2016**	8.1 (±1.6)	8.0 (±1.5)	0.957	41.0 (±5.2)	39.0 (±6.4)	0.182	2.11 (±0.53)	2.08 (±0.56)	0.328
**Hadimeri et al, 1998**	-	-	-	-	-	-	-	-	-
**Koc et al, 2016**	10.7 (±2.0)	10.3 (±2.0)	0.38	38.6 (±5.1)	35.6 (±6.6)	0.03*	2.15 (±0.40)	2.03 (±0.60)	0.47

PKD+, *Patients with Polycystic Kidney Disease*; PKD-, *Patients without Polycystic Kidney* Disease; Kt/V, Total *weekly Kt/V urea*

*, p<0.05

### Outcomes

#### Overall survival

**Fig 2:**
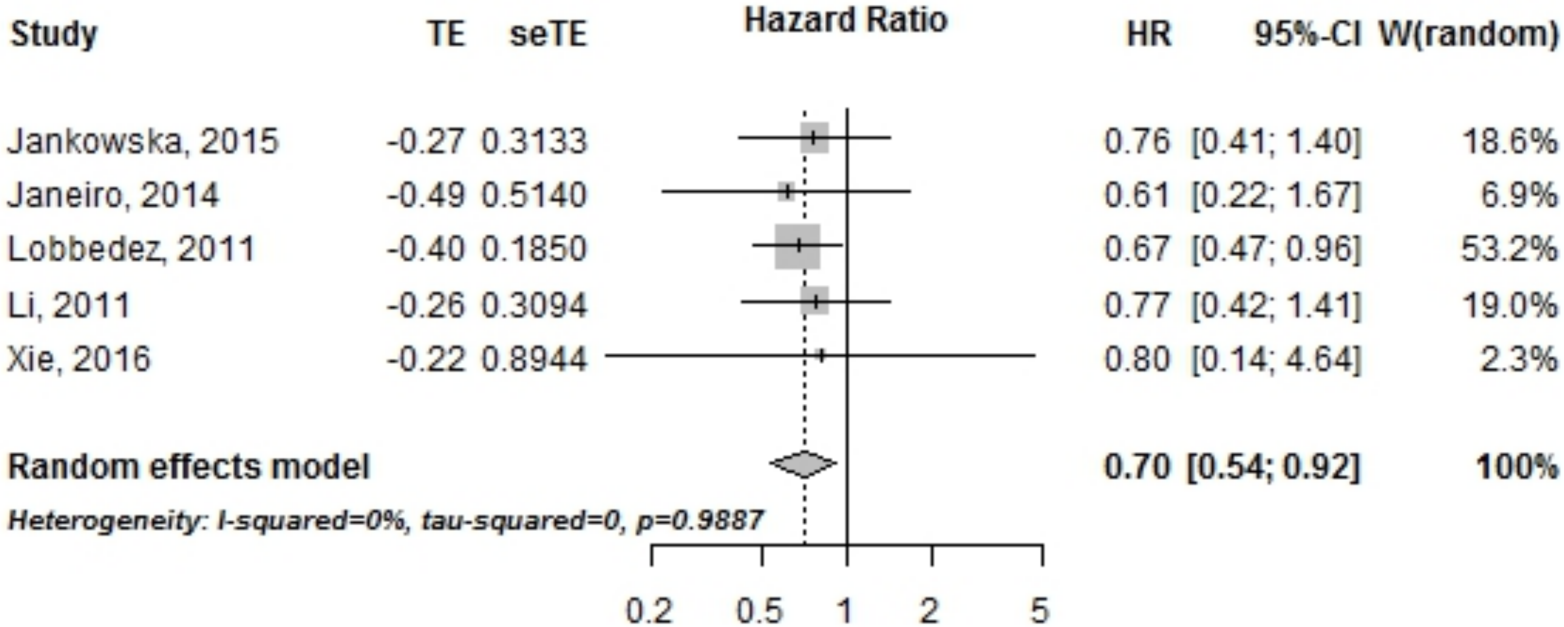
Overall survival of PKD vs non PKD patients treated by peritoneal dialysis. HR, *Hazard ratio*; CI, *Confidence interval*.

There were 5 studies that reported hazard ratios for PKD and non PKD patient survival. 6,378 patients from Europe and China were included in the meta-analysis. PKD status was associated with a better global survival with a Hazard Ratio of 0.70 [95% CI, 0.54–0.92] (Fig 2).

#### PD technique survival

**Fig 3:**
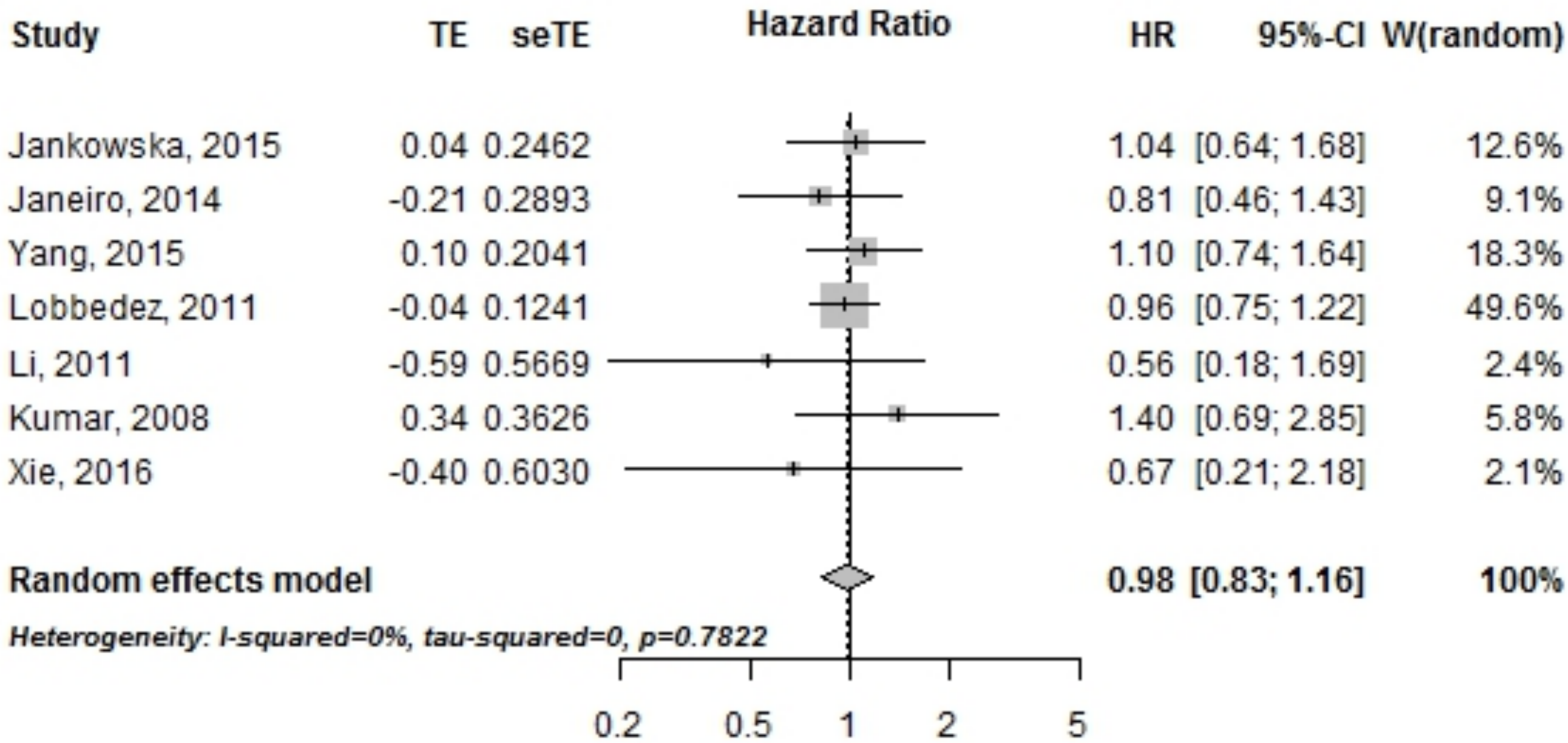
Peritoneal dialysis technique survival in PKD vs non PKD patients. HR, *Hazard ratio*; CI, *Confidence interval*.

Seven studies reported hazard ratios for PD technique survival in both groups, which included a total of 7,046 patients from Europe, China and Taiwan. There was no difference between PKD and non PKD groups in terms of PD technique survival (HR = 0.98 [95% CI, 0.83–1.16]) as shown in Fig 3.

#### Infectious complications

**Fig 4:**
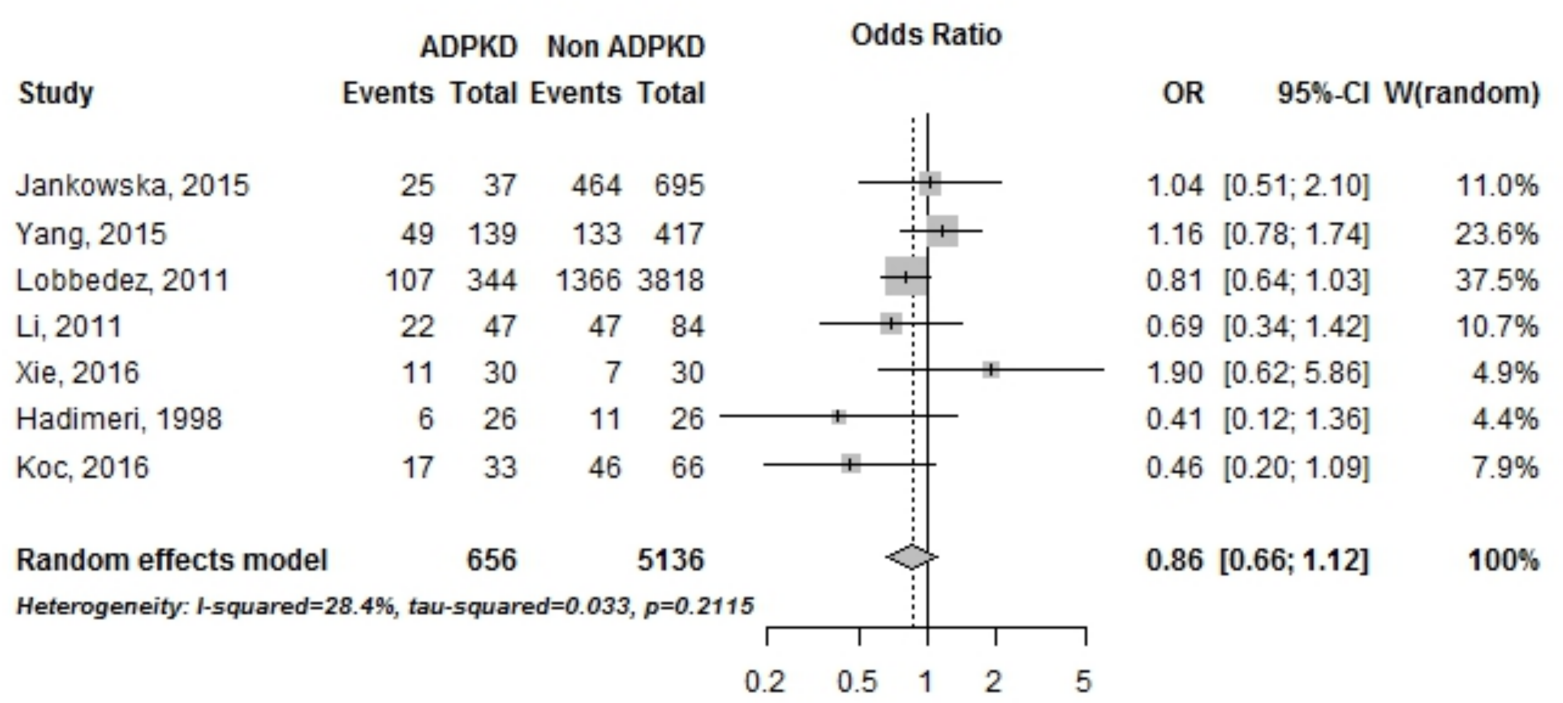
Incidence of infectious peritonitis in PKD vs non PKD patients treated by peritoneal dialysis. OR, *Odds ratio*; CI, *Confidence interval*.

Seven studies reported odds ratios for the incidence of infectious peritonitis episodes in PKD and non PKD patients treated by PD. 6,767 patients from Europe, China, Taiwan and Turkey were included in the meta-analysis (Fig 4). There was no statistical difference in occurrence of infectious peritonitis between PKD and non PKD patients (OR = 0.86 [95% CI, 0.66–1.12]) as shown in Fig 4.

#### Abdominal wall hernias

**Fig 5:**
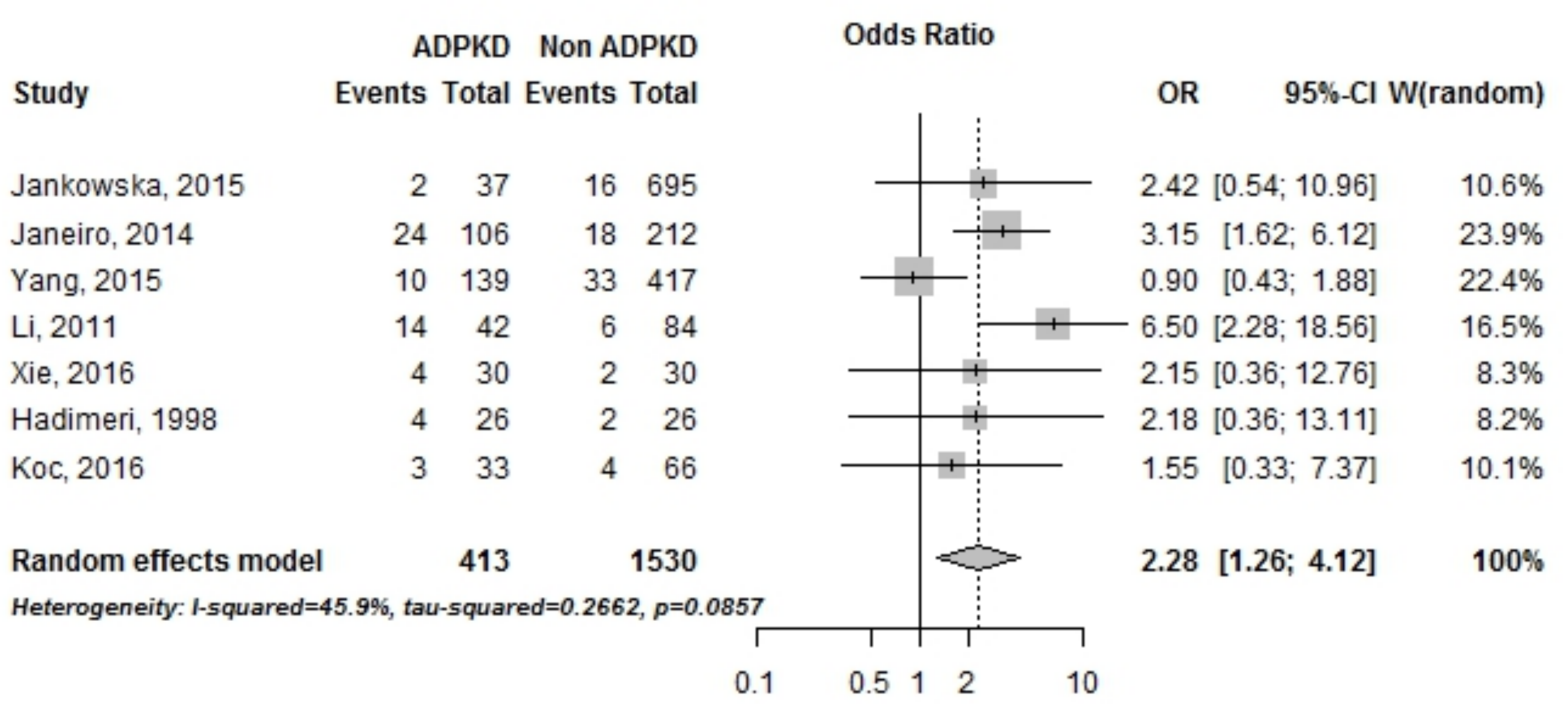
Incidence of hernias in PKD vs non PKD patients treated by peritoneal dialysis. OR, *Odds ratio*; CI, *Confidence interval*.

There were 7 studies that reported odds ratios for the incidence of abdominal wall hernias in PKD and non PKD patients treated by PD, including a total of 2,923 patients from Europe, China, Taiwan and Turkey (Fig 5). PKD patients were found to have an increased risk of abdominal hernia with an Odds Ratio of 2.28 [95% CI, 1.26–4.12] as shown in Fig 5.

#### Sensitivity analysis

Because the large weight of the study by Lobbedez et al. among the included studies might affect the entire analysis, we performed sensitivity analysis to check the stability of the previous results without including the study by Lobbedez et al. (except for abdominal wall hernias because this study was not included in analysis for this outcome). Results are summarized on Table 5. Hazard ratios and Odds ratios showed a great stability even in the absence of the study by Lobbedez et al. The only observed change in the absence of the study by Lobbedez et al. was the absence of statistical relevance in the analysis of overall survival.

**Table 5:** Sensitivity analysis for overall survival, PD technique survival and infectious complications with or without Lobbedez et al. study.

	Hazard ratio or Odds Ratio	Confidence interval	Hazard ratio or Odds Ratio w/o Lobbedez et al.	Confidence interval w/o Lobbedez et al.
**Overall survival**	HR = 0.70	0.54–0.92	HR = 0.74	0.5–1.09
**PD technique survival**	HR = 0.98	0.83–1.16	HR = 1.00	0.79–1.27
**Infectious complications**	OR = 0.86	0.66–1.12	OR = 0.87	0.59–1.28

w/o, *without*; HR, *Hazard ratio*; OR, *Odds ratio*

## Discussion

To our knowledge this is the first meta-analysis designed to study the outcome of PKD patients on PD. We found that compared to non PKD patients, PKD patients on PD had i) better global survival, ii) no difference in PD technique survival, iii) no difference in peritonitis rate but increased risk of abdominal hernias. Our results should reassure nephrologists managing PKD patients regarding the choice of PD as renal replacement therapy.

We found that PKD patients had better survival compared with non PKD patients. This difference could be explained by lower comorbidities reported in PKD patients: younger age [14,20], lower Charlson index [14] and higher hemoglobinemia [14,17]. On the other hand, patients with this hereditary disease are usually diagnosed earlier and monitored with a better nephrology care and a planned access to PD in optimized conditions. Only one study reports this factor but it could certainly explain at least in part the good clinical outcome for PD group in other studies [14].In addition, PKD patients have less comorbidities and may therefore have better outcome and a relatively lower risk of death or transfer to hemodialysis because of a higher competing risk of being transplanted (Table 3). Lastly, in one study, diabetes mellitus was found to be the only predictor of all-cause mortality independently from PKD status [13].

Furthermore, the meta-analysis strategy allowed us to include 7,046 patients for the analysis of PD technique survival. PD technique survival did not differ according to PKD status. Moreover, there were no statistical difference between PKD and non PKD patients in terms of access to kidney transplantation or transfer to hemodialysis. Koc et al. reported that causes of death and transfer to hemodialysis were not different between PKD and non PKD patients (p = 0.35 and 0.36, respectively) [16]. In the study of Kumar et al, in multivariate analysis [18], hypoalbuminemia at initiation of PD was found to be the main risk factor for PD therapy cessation independently from PKD status.

Previous reports have suggested an increased incidence of mechanical complications in PKD patients treated with PD, such as abdominal leak or hernia [5,12]. In these studies, abdominal hernias were not associated with an increased risk of PD discontinuation. In the present meta-analysis, we confirm that episodes of abdominal hernia appeared statistically more often in PKD patients treated with PD. However, abdominal wall complications have been found to be more frequent in PKD patients at all stages of kidney disease including before end stage renal disease. As a consequence, it is likely that hernias may not be directly related to increased intraperitoneal pressure but may be related to collagen defects and thus be observed in PKD patients treated with other renal replacement therapies [21]. Increased risk of abdominal hernias was not associated with a decrease in technique survival. This suggests that for these patients, the therapeutic intervention such as reducing intraabdominal pressure (reduced volume infused of peritoneal solution or using automated peritoneal dialysis) is successful for maintaining them on PD therapy. In addition, studies included in this meta-analysis did not show any difference in term of fluid leak episode incidence [13,14,18,20].

Some reports indicate that PKD may be associated with an increased risk of diverticulitis, leading to greater risk of peritoneal infection in patients treated with PD [5]. In the present meta-analysis, including 6,767 patients, we found that PKD was not associated with a higher risk of occurrence of peritoneal infection. Furthermore, incidence of peritonitis episode requiring catheter removal did not differ between groups in Kumar et al. and Xie et al. (25% in PKD group vs 21% in non PKD group and 6,7% in PKD group vs 3,3% in non PKD group respectively) [18,19]. Staphylococcus *spp* was the main causative micro-organism in both groups while gram-negative organisms incidence did not differ between PKD and non PKD patients [14,15,17,18,19].

PD treatment offers numerous advantages [22,23], however only 7 to 10% of end-stage renal disease patients are treated with PD [2]. This reflects, at least in part, the clinicians’ fear of technical failure [24]. PKD patients are probably more concerned by this issue, because of the common misconception that these patients will develop infectious and mechanical complications if treated with PD, due to increased intraperitoneal pressure and a higher incidence of diverticulitis. Our results suggest that PD is a safe renal replacement modality for PKD patients. To the best of our knowledge, there is no available study designed to assess the impact of different PD modalities (continuous ambulatory peritoneal dialysis or automated peritoneal dialysis) on PKD patients’ outcomes.

First limitation of our study is related to the small number of publications on this subject. Additionally, all the studies included in the meta-analysis are retrospective or registry based studies. Indeed, there is no randomized clinical trial available on this subject. Therefore, a potential selection bias may limit the relevance of our conclusions for the entire PKD population. However, we collected all the study available (ie 9) and a total of 7,197 patients across the 9 studies (n = 2,923 to 7,046 patients for each outcome). This was a sufficient prerequisite to perform a meta-analysis. Another limitation is related to the relative importance of the study by Lobbedez et al. among the included studies due to the large number of patients included (n = 4162) accounting for almost half of the total population and Furthermore the design of the Lobbedez study, was different it was the only one including survival analysis after exclusion of the diabetic patients. However our results show strong stability when analyzed with or without Lobbedez’s study.

In conclusion, this meta-analysis showed that PKD patients treated with PD seem to have an increased survival and an increased rate of abdominal hernia, without any impact on PD technique survival. There was no statistical difference in peritonitis rate between PKD and no PKD patients. Therefore, our data suggest that peritoneal dialysis is a safe modality to treat end stage renal disease of PKD patients and should be offered to these patients. However, properly designed controlled studies are needed to determine whether all PKD patients are eligible for PD or whether some specific criteria should be determined. A particular attention should be given to the impact of total kidney volume and liver size in the feasibility of PD.

## Supporting information

S1 Checklist PRISMA checklist. (DOC)
